# Pre-clinical indications of brain stimulation treatments for non-affective psychiatric disorders, a status update

**DOI:** 10.1038/s41398-023-02673-2

**Published:** 2023-12-14

**Authors:** Lindsay L. Benster, Cory R. Weissman, Louise A. Stolz, Zafiris J. Daskalakis, Lawrence G. Appelbaum

**Affiliations:** 1 Joint Doctoral Program in Clinical Psychology, SDSU/UC San Diego, San Diego, CA USA; 2 Department of Psychiatry, UC San Diego School of Medicine, San Diego, CA USA

**Keywords:** Psychiatric disorders, Psychology

## Abstract

Over the past two decades noninvasive brain stimulation (NIBS) techniques have emerged as powerful therapeutic options for a range of psychiatric and neurological disorders. NIBS are hypothesized to rebalance pathological brain networks thus reducing symptoms and improving functioning. This development has been fueled by controlled studies with increasing size and rigor aiming to characterize how treatments induce clinically effective change. Clinical trials of NIBS for specific indications have resulted in federal approval for unipolar depression, bipolar depression, smoking cessation, and obsessive-compulsive disorder in the United States, and several other indications worldwide. As a rapidly emerging field, there are numerous pre-clinical indications currently in development using a variety of electrical and magnetic, non-convulsive, and convulsive approaches. This review discusses the state-of-the-science surrounding promising avenues of NIBS currently in pre-approval stages for non-affective psychiatric disorders. We consider emerging therapies for psychosis, anxiety disorders, obsessive-compulsive disorder, and borderline personality disorder, utilizing transcranial magnetic stimulation (TMS), transcranial direct current stimulation (tDCS), and magnetic seizure therapy (MST), with an additional brief section for early-stage techniques including transcranial focused ultrasound stimulation (tFUS) and transcranial alternating current stimulation (tACS). As revealed in this review, there is considerable promise across all four psychiatric indications with different NIBS approaches. Positive findings are notable for the treatment of psychosis using tDCS, MST, and rTMS. While rTMS is already FDA approved for the treatment of obsessive-compulsive disorder, methodologies such as tDCS also demonstrate potential in this condition. Emerging techniques show promise for treating non-affective disorders likely leading to future regulatory approvals.

## Introduction

Brain stimulation is a rapidly evolving field of clinical medicine and neuroscientific research. Noninvasive brain stimulation (NIBS) approaches that modulate neuronal activity through external magnetic or electrical fields have emerged as a “third branch” of psychiatric medicine alongside psychotherapy and pharmacotherapy [1]. Currently, NIBS approaches span an ever-increasing collection of methodologies that aim to safely alter brain function and induce therapeutic change in the brain to combat psychiatric and neurological illness [1]. Stimulation techniques include both invasive and noninvasive approaches that are capable of producing either excitation or inhibition of functional circuits or specific regions, depending on the stimulated area, stimulation parameters (frequency and pattern), disease state of underlying tissue, and polarization state of targeted neurons [2]. Modalities include convulsive procedures, such as electroconvulsive therapy (ECT) and magnetic seizure therapy (MST), which aim to safely induce therapeutic seizures, as well as a wide array of non-convulsive and sub-convulsive therapies capable of evoking brain changes in the absence of seizures [3, 4]. In addition to these more widely-used techniques, there is growing excitement surrounding novel forms of stimulation, such as transcranial focused ultrasound (tFUS), in which short bursts of low-intensity sonic waves affect voltage-dependent neural processes in the brain [5]. Collectively, this diversity of stimulation techniques and parameters offers unique opportunities to target dysregulated brain networks distributed widely throughout the brain.

While classic treatments for psychiatric disorders typically include combinations of pharmacotherapies and psychotherapies, a large proportion of individuals do not successfully respond to these interventions [6]. An estimated 30–40% of individuals with major depressive disorder (MDD) do not respond to the first two antidepressant treatments, roughly 50% of individuals with generalized anxiety disorder do not respond to first-line treatments, 25–50% of individuals with schizophrenia experience auditory hallucinations that persist despite antipsychotic medication treatment, and 10–40% of patients with obsessive-compulsive disorder (OCD) experience treatment resistance [2–5, 7, 8]. Neuromodulatory techniques, therefore, hold promise to augment and potentially replace pharmacologic and psychotherapeutic approaches and improve patient outcomes when primary, and often secondary, interventions fail.

To date, NIBS has primarily been studied to treat mood disorders such as MDD and demonstrates considerable success inducing remission in treatment-resistant individuals and normalizing aberrant connectivity in disrupted neural networks. For instance, recent innovations in transcranial magnetic stimulation (TMS) technology have led to more rapid and potent stimulation protocols such as intermittent theta burst stimulation (iTBS) and accelerated protocols [9, 10]. These forms of TMS involve condensed treatment schedules with high-frequency bursts of stimulation that entrain endogenous brain rhythms, leading to greater neuroplasticity and reduction of symptoms in shorter timeframes. Trials implementing accelerated iTBS protocols have yielded remission rates up to 90% in patients with MDD [10] and were recently FDA approved for the treatment of depression [11]. Due to the growing success of rTMS and other NIBS for MDD, these modalities have been piloted for the treatment of nonaffective disorders.

The goal of this article is to review the current state-of-the-science of NIBS techniques that are in development for the treatment of psychiatric diseases. Given the substantial evidence for NIBS in the treatment of mood disorders, in this review we explore their potential use in nonaffective conditions that have not yet received federal approval: psychosis, anxiety, obsessive-compulsive disorder, and borderline personality disorder. Neurodevelopmental disorders such as attention deficit hyperactivity disorder (ADHD) and autism spectrum disorder (ASD) were excluded due to theorized differences in pathophysiology. The first section of this review introduces the psychopathology of each included nonaffective disorder, then an overview of the included NIBS aproaches is provided, which is followed by a review of the current state-of-research for NIBS therapeutics for each indication. Table 1 includes all acronyms used throughout this review.

**Table 1:** Acronyms.

Acronym	Definition	Acronym	Definition
ACC	Anterior cingulate cortex	NIBS	Noninvasive brain stimulation
BPD	Borderline personality disorder	OCD	Obsessive-compulsive disorder
CBT	Cognitive behavioral therapy	OFC	Orbitofrontal cortex
cTBS	Continuous theta burst stimulation	PANSS	Positive and negative symptom scale
DBS	Deep brain stimulation	PD	Panic disorder
DLPFC	Dorsal lateral prefrontal cortex	PTSD	Post-traumatic stress disorder
DMPFC	Dorsomedial prefrontal cortex	RCT	Randomized controlled trial
ECT	Electroconvulsive therapy	ROI	Region of interest
ERP	Exposure with response prevention therapy	rTMS	Repetitive transcranial magnetic stimulation
FDA	Food and drug administration	SAD	Social anxiety disorder
fMRI	Functional magnetic resonance imaging	SGC	Subgenual cingulate cortex
GAD	Generalized anxiety disorder	SMA	Supplementary motor area
HPA	Hypothalamic-pituitary adrenal	SSRI	Selective serotonin reuptake inhibitor
iTBS	Intermittent theta burst stimulation	tACS	Transcranial alternating current stimulation
LTD	Long-term depression	tDCS	Transcranial direct current stimulation
LTP	Long-term potentiation	tFUS	Transcranial focused ultrasound
MDD	Major depressive disorder	TMS	Transcranial magnetic stimulation
mPFC	Medial prefrontal cortex	TPJ	Temporal parietal junction
MST	Magnetic seizure therapy	TRD	Treatment-resistant depression

## Methods

The methodology employed in this scoping review followed the framework outlined by Arksey and O’Malley (2005) [12]. The framework consisted of five notable steps: 1. Formulating the research question, 2. Identifying pertinent studies, 3. Selecting relevant studies, 4. Organizing the data, and 5. Combining, summarizing, and discussing the findings.

### Research question

This review was guided by the question: “What is the current state of NIBS for psychiatric disorders that do not yet have federal approval?” Due to the convincing literature for FDA-approved indications that are currently implemented in clinical practice, this review aims to discuss the status of NIBS for possible indications in the pre-approval stages. These include: psychosis (primarily schizophrenia), anxiety disorders, trauma and stress-related disorders, obsessive-compulsive disorder, and borderline personality disorder. These particular disorders were included due to strong overlap in abnormal circuitry associated with symptom expression. Other recognized psychiatric disorders, such as neurodevelopmental, personality, and substance use disorders, were excluded due to likely differences in neural network involvement and etiology.

### Search strategy

The initial searches were conducted between November 2021 and March 2022. Follow-up searches were conducted in July 2023. This began with the identification of pertinent terms, including specific searches for each modality in the following electronic databases MEDLINE/PubMed, PsycInfo, and EMBASE: “brain stimulation”; “transcranial magnetic stimulation”; “transcranial direct current stimulation”; “deep brain stimulation”; “electroconvulsive therapy”; “magnetic seizure therapy”; “psychiatric disorders”; “mental disorders.” Searches for specific subsections were also conducted and reference lists of reviews were additionally consulted.

### Study selection

Systematic reviews and meta analyses received preferential consideration. Articles solely considering brain stimulation techniques in animal models, non-English languages, or non-psychiatric disorders were excluded. Moreover, only adult populations were considered. Depression was included in the tFUS section due to the novelty of the modality and lack of existing research convincingly demonstrating its efficacy.

### Data organization

The process of organizing the data encompasses essential procedures for how information is extracted from the primary sources reviewed. Here, a narrative review approach that contextualizes outcomes for enhanced understanding was used. While specific details of each individual study are not provided, general findings are condensed across studies to contribute to a comprehensive narrative.

### Combining, summarizing, and discussing results

In line with the aims of a scoping review, the overall aim of this manuscript is not necessarily to evaluate the quality of studies and synthesize across eligible studies as in a systematic review. The purpose instead revolves around identifying general findings and significant gaps that warrant further exploration.

## Nonaffective psychiatric disorders

The psychiatric diagnoses discussed in this review broadly encompass nonaffective and pre-approved disorders in NIBS, including psychotic disorders (i.e., schizophrenia), anxiety disorders (i.e., generalized anxiety disorder (GAD), social anxiety disorder (SAD)), trauma disorders (i.e., post-traumatic stress disorder (PTSD)), OCD, and borderline personality disorder (BPD). Affective disorders, or mood disorders, are a group of psychiatric disorders characterized by chronic abnormalities of emotional state. Nonaffective disorders involve DSM-5 recognized conditions in which a disruption in emotional state is not the primary symptom. The selected nonaffective disorders included here are highly heterogenous in their symptomology and affected neural mechanisms, which leads to high rates of treatment resistance and a profound need to develop more effective therapies [6]. Nuances in their symptom presentation and abnormal connectivity highlight important considerations for implementation of interventions. The first group of disorders which will be discussed in this review is psychosis. The second group of disorders discussed is anxiety and traumatic stress-related disorders. Although anxiety disorders cover a broad array of pathologies, they have underlying common disruptions in neural pathways which may be amenable to neuromodulation therapy. Third, OCD is diagnostically classified as a separate family of disorders than anxiety, despite notable overlap in the underlying pathology and involved circuits. Finally, BPD is another nonaffective illness that consists of disrupted emotion processing and behavioral dysregulation. Although these nonaffective disorders have highly variable symptom profiles, their pathology broadly involves dysregulation of the limbic and frontal networks, both of which have been successful treatment targets for NIBS in mood disorders.

Importantly, the disease burden of psychotic, anxiety, and borderline personality disorders remain extensive. Schizophrenia is considered one of the top 15 leading causes of disability across the globe, placing individuals at increased risk for premature mortality, co-occurring medical conditions, and requiring high demand of financial and social resources [13, 14]. Anxiety disorders are common with approximately 275 million individuals affected worldwide, representing high economic burden and widespread impact [15]. Improving treatment options and accessibility are critical to alleviating disease burden of all psychiatric conditions. NIBS provides a unique opportunity to potentially reduce burden significantly across these conditions by inducing exogenously produced, controllable neuroplasticity to counteract pathology with greater specificity, fewer side effects, and less burden than standard treatments [16].

## Overview of brain stimulation approaches

To best illustrate the current state of the field, this review focuses on NIBS for the treatment of nonaffective psychiatric disorders, highlighting the areas with the most promise, and identifying outstanding gaps in the literature. Three main forms of NIBS with various depths of supportive evidence and their side effect profiles are discussed. These include: transcranial direct current stimulation (tDCS), TMS, and MST. An additional section describes the status of emerging modalities (tFUS and tACS).

The therapeutic use of TMS relies on repeated magnetic pulses and is referred to as repetitive TMS (rTMS). Over the last several decades, rTMS treatment parameters have been modified for enhanced clinical utility across a range of disorders [17]. During TMS treatment, a coil placed over the scalp generates a very brief (~4 ms) magnetic field that passes through tissues with low electrical conductivity such as skin, bone, and fat, with little deflection, and causes rapid neuronal depolarization in cortical tissue. Field penetration is limited, with decreasing intensity as the distance from the center of the coil increases. Depending on the coil type, the peak field occurs at depths ranging from about 1 to 4 centimeters [18]. While typical clinical use of TMS involves placement of coil location based on scalp distances, fMRI and neuronavigation technologies now allows for targeting that is specific to the neurocircuitry of each patient [19]. Moreover, TMS can be applied either ‘offline’ as indviduals rest without active cognition, or ‘online’ during structured cognitive tasks, leading to applications and research with both approaches [20]. rTMS protocols can also be categorized into high or low frequency. High-frequency rTMS typically involves the delivery of magnetic pulses at frequencies at or greater than 1 Hz [21]. This protocol typically has an excitatory effect on cortical activity, and is often used to target regions associated with hypoactivity, or to modulate neural circuits involved in various psychiatric and neurological disorders. In contrast, low-frequency rTMS employs frequencies at 1 Hz or below. This typically produces inhibitory effects on cortical excitability and is often used to target regions associated with hyperactivity. The duration and frequency of TMS treatment varies considerably across studies. Many treatments administer one session per day for one to six weeks, while newer accelerated therapies administer up to 10 sessions per day for five days [10]. These accelerated protocols aim to provide more condensed treatment courses, potentially reducing the overall duration of treatment while maintaining therapeutic efficacy. One such protocol that is noteworthy to mention is the application of accelerated continuous/intermittent theta burst stimulation (TBS). Continuous TBS (cTBS) involves the delivery of bursts of magnetic stimulation at a frequency of 50 Hz, repeated every 200 milliseconds for a total duration of 40 s. Intermittent TBS (iTBS) delivers bursts at the same frequency but follows an intermittent pattern, with bursts delivered every 10 s for a total duration of approximately 190 s or around 600 pulses. Accelerated iTBS sessions involve a duration of 3 min as opposed to 30 min for rTMS, and multiple sessions can be administered within the same day.

TDCS involves transmission of low-amplitude (<2 mA) electrical currents that propagate between electrodes that are placed on the scalp. Stimulation through an anodal electrode (positive current) has been shown to increase excitability of underlying neurons while stimulation through the cathodal electrode (negative current) has been shown to decrease excitability [7]. The effects of tDCS depend largely upon intervention parameters, such as the duration of stimulation, the positioning of the electrodes, and whether the stimulation is delivered during a concurrent task or not [22]. In the case of tDCS, the delivered current does not directly generate action potentials, rather it facilitates or inhibits synaptic transmission by increasing or decreasing neuronal polarization which, in turn, leads to changes in long-term potententation or depression (LTP/LTD) [1]. Relatedly, transcranial alternating current stimulation (tACS) is a similar form of electrical current stimulation in which the polarity of the stimulation alternates according to a sinewave function to drive activity at a desired frequency [23].

Less research has been conducted on MST compared to other forms of neuromodulation. MST involves inducing a therapeutic seizure using high-powered rTMS. As a focalized form of convulsive therapy, MST aims to replicate the efficacy of ECT, while reducing side effects, such as cognitive deficits [24–26]. During MST, high intensity magnetic pulses are delivered through a TMS coil to induce a seizure. Like rTMS, the alternating magnetic fields produced during MST penetrate the skull with minimal resistance, limiting the spread of the induced seizure and increasing precision [24]. In preliminary studies, MST has demonstrated promise as a clinical tool for MDD with a current confirmatory non-inferiority clinical trial underway comparing MST to ECT [24, 25, 27, 28].

Collectively, NIBS appear to normalize aberrant activity in affected brain regions in a safe and potentially less burdensome manner compared to traditional therapeutics. The remainder of this review will discuss the studies that have been conducted thus far on nonaffective disorders using these modalities.

## Review of indications

### Psychosis

Psychotic disorders encompass schizophrenia spectrum and related disorders, which are characterized by abnormalities in at least one of the following five symptom domains: delusions, hallucinations, disorganized thinking, grossly disorganized or abnormal motor behavior, or negative symptoms, such as flat affect, reduced avolition and disorganized thought, disrupted speech, and behavior [29, 30]. Schizophrenia is the most commonly diagnosed and studied psychotic disorder, with an estimated prevalence of 2.8 million adults in the United States [31, 32]. Importantly, schizophrenia represents one of the more commonly studied disorders using NIBS, however many clinical trials have attempted to analyze the effects of neuromodulation on both positive and negative symptoms, yielding inconsistent results [33]. Although the neuropathology underlying these illnesses has not been fully elucidated, dysregulation of the limbic system and the frontoparietal cortex is commonly reported [29]. Moreover, the mechanisms underlying positive and negative symptoms in schizophrenia differ, so results from clinical trials will be discussed for each group of symptoms separately. First-line treatment for schizophrenia typically involves antipsychotic medications often augmented with psychotherapeutic intervention [34]. Antipsychotic medications have been largely effective in reducing the experience of positive symptoms, but are less effective at reducing negative symptoms, disorganization, and cognitive dysfunction [35]. At present, treatment regimens for schizophrenia are limited; in addition to the estimated 30% treatment resistance, interventions produce numerous adverse effects, inadequately treat the wide spectrum of symptoms, and continue to be associated with adherence challenges. Therefore, there is great interest in the capability of NIBS to combat psychotic symptoms that are notoriously difficult to treat. Evidence suggests abnormal hyper-connectivity between the dorsal lateral prefrontal cortex (DLPFC) and the temporal parietal junction (TPJ) may be implicated in the experience of auditory hallucinations [36, 37]. As such, NIBS could potentially target this abnormal activity by inducing LTD between these overactive networks through inhibitory stimulation protocols. However, the literature is replete with inconsistent findings and thus much more study is needed before conclusions can be drawn. Neuroimaging studies have demonstrated the involvement of DLPFC hypofunctioning and cortico-subcortical circuits in negative and cognitive symptoms. Reduced activation in the DLPFC during working memory tasks has been observed in individuals with more severe negative symptoms, suggesting a connection between DLPFC dysfunction and presenting symptoms [38]. Interactions between the DLPFC and other regions, such as the striatum and thalamus, are also implicated in the emergence of cognitive and negative symptoms. These findings provide potential targets for NIBS in alleviating hypofunctioning of brain regions, and restoring associated cortico-subcortical circuits.

#### Transcranial magnetic stimulation

TMS has been utilized to treat positive symptoms of schizophrenia, such as persistent auditory hallucinations, as well as cognitive deficits, and negative symptoms. This has been driven by the unique capabilities of rTMS to induce neurobiological changes related to specific neuropathology underlying schizophrenia [39]. In these studies, rTMS is administered to the left PFC. Meta-analyses suggest small-to-modest effect sizes (0.29–0.64) for the benefits of rTMS over sham treatment for negative, positive, and cognitive symptoms [37]. Notably, certain factors are associated with greater rTMS response in psychosis: younger age, female gender, higher prescribed antipsychotic dosage, increased cerebral blood flow at stimulation site, and shorter scalp-to-temporal cortex distance [39–41].

Auditory hallucinations are the primary positive symptom that has been studied with rTMS. Low-frequency rTMS can reduce cortical hyperactivity involved in auditory hallucinations [38]. A systematic review of 30 rTMS studies targeting positive symptoms found evidence that rTMS reduced auditory hallucinations in half of the studies [41]. However, an additional systematic review and meta-analysis found rTMS ineffective in treating positive symptoms, but effective in treating negative and cognitive symptoms [42]. These protocols targeting hallucinations primarily administered low-frequency rTMS to the left TPJ, although stimulation sites and parameters varied widely, rendering interpretation of the evidence for the use of rTMS to reduce positive symptoms challenging. Further study in this area is warranted.

Negative symptoms of schizophrenia (anhedonia, affective flattening, social withdrawal, avolition) are believed to result from a decrease in meso-cortical signaling. As such, most studies targeting such brain areas delivered high-frequency rTMS to the left or bilateral PFC. Significant decreases in negative symptoms following rTMS interventions have been seen, exemplified through a randomized, double-blind, sham-controlled trial (*N* = 60) administering 4-weeks of 20-Hz rTMS to the DLPFC [39]. These improvements have not extended to cognition in most studies, although one meta-analysis reports positive effects of rTMS on working memory, but no other cognitive domain [43]. A meta-analysis and systematic review corroborated these findings, providing evidence for combining rTMS with antipsychotic treatment for slight improvements in negative symptoms [40]. Importantly, included studies were highly heterogeneous and therefore conclusions must be cautiously examined.

#### Transcranial direct current stimulation

tDCS for psychosis targets auditory hallucinations or negative symptoms by increasing cortical excitability of the TPJ and decreasing cortical excitability of the DLPFC. It is hypothesized that abnormalities in connections between these two regions drive symptoms [44]. Electrode montages generally implemented in clinical trials typically involve one of the following: 1. Targeting aberrant tempopariental hyperactivity responsible for auditory hallucinations with cathodal stimulation coupled with anodal stimulation on the hypoactive prefrontal cortex; or 2. Bilateral stimulation of the prefrontal cortex, aiming for improvement of negative and cognitive symptoms through targeting hypoactivity in the DLPFC. Moreover, unilateral left bipolar-tDCS with anodal placement over the DLPFC and cathodal placement over the TPJ, may result in auditory hallucinations and positive symptom improvements, possibly through frontal hypo-functional and parieto-temporal hyperactivity restorations [45, 46]. Conversely, bilateral-bipolar prefrontal stimulation with anodal placement over the left DLPFC and cathodal contralaterally placed over other frontal areas, may improve negative symptoms and cognition. Several double-blind sham-controlled randomized controlled trials (RCTs) support the ability of tDCS to mitigate psychotic symptoms in schizophrenia and schizoaffective disorder, particularly through reductions in these two clinical features [37, 44, 47, 48–51]. The most common arrangement places electrodes over the fronto-temporal network [37, 44, 47].

Studies implementing tDCS for treatment-resistant positive symptoms have delivered treatment to the anodal left DLPFC and cathodal left TPJ stimulation across 10 sessions, yielding significant reductions in reported auditory hallucinations compared to sham with a mean diminution of 31%, and effects persisting up to three months post treatment [15–18]. Despite some successes, not all studies have been effective in ameliorating auditory hallucinations [52]. However, stimulation parameters did differ in terms of frequency of stimulation across studies (twice daily for five days compared to once daily for three weeks). While most studies support the utility of tDCS for the treatment of auditory hallucinations in psychotic disorders, negative findings highlight the importance of refining intervention designs and the schedule of stimulation. Targeting of disrupted circuitry between TPJ and DLPFC appears to be effective, supporting theories of mechanisms implicated in auditory hallucinations.

Several studies have examined the reduction of negative symptoms solely or conjointly with auditory hallucinations [47, 51, 53]. Such studies have found significant reductions in symptoms such as emotion processing, mood impairments, and cognitive control, from targeting prefrontal areas [42, 47, 54–57]. Studies examining negative symptoms alone have found significant improvements following tDCS specifically in passive/apathetic withdrawal, expressive deficits, stereotyped thinking, psychosocial functioning, disorganization, and overall cognitive deficits [53, 56, 57]. Negative symptom improvement could be attributed to the stimulation of cortical-subcortical networks, and interestingly, medications were also found to influence the efficacy of treating negative symptoms [56]. Haloperidol and clozapine decreased tDCS effects potentially due to their influence of plasticity and its eliminatory effect on anodal excitation [53].

With regards to cognitive symptoms, tDCS has been shown to improve working memory deficits supported by 12 out of 18 studies in a systematic review. However, several metaanalyses have indicated that the variation in stimulation parameters may account for inconsistent results between groups [56, 58–60]. Additionally, studies analyzing the effect of tDCS on cognitive symptoms as a secondary outcome found no improvements compared to sham in cognition including executive function, memory, and social cognition. Although in early stages, tDCS demonstrates potential for treating auditory hallucinations and negative symptoms in psychotic disorders, with opportunities to explore additional clinical features. Further study exploring how tDCS compares to existing first-line interventions are needed.

#### Magnetic Seizure Therapy

Relatively few studies have examined treatment of psychosis and related disorders with MST. Two pilot and feasibility studies in patients with schizophrenia or treatment-resistant schizophrenia (*N* = 8 in both studies) have found significant clinical and quality-of-life improvements following intervention. These include response rates of 50% or more in the reduction of depressive symptoms following 10 sessions of MST over four weeks, without significant adverse cognitive effects [61, 62]. A larger study examining the efficacy of MST compared to ECT in patients with schizophrenia (*N* = 79) found that MST yielded a non-significant larger antipsychotic effect measured via positive and negative symptom scale (PANSS) reduction and response [63]. Importantly, MST did produce significantly less cognitive impairment compared to ECT as measured by immediate memory, language, delayed memory, and global cognitive function. Similar efficacy with fewer adverse effects minimizes patient burden and improves palatability of treatment, rendering MST a promising alternative to ECT for treatment of schizophrenia. However, the identified studies did indicate a relatively high rate of discontinuing MST treatment for a variety of reasons, primarily dizziness and subjective report of memory loss [64]. Further studies with larger sample sizes are necessary to explore MST for treatment-resistant psychotic disorders, particularly in non-depressive symptoms.

### Anxiety and stressor-related disorders

Anxiety disorders share elements of excessive fear, anxiety, and behavioral disturbances [65]. The DSM-5 includes several distinct categorizations, but the most studied in neurostimulation research include GAD, SAD, panic disorder (PD), and agoraphobia. Trauma and stress-related disorders (PTSD) are additionally included in this section due to high degree of overlap and comorbidity. Approximately 19.1% of adults in the US are estimated to experience an anxiety disorder [66]. Standard treatment of anxiety disorders incorporates antidepressant medications, particularly selective serotonin reuptake inhibitors (SSRIs), and cognitive behavioral therapy (CBT). While effective in many individuals, a considerable proportion of people do not benefit from these treatments and NIBS may therefore offer an effective alternative.

Many of the common clinical features of anxiety-related disorders arise from disrupted meso-cortico-limbic circuitry [67]. Anxiety disorders are characterized by multiple disruptions in the limbic system and cognitive-emotional neural networks, leading to overactivation of the hypothalamic-pituitary adrenal axis (HPA), the amygdala, which controls the fear response, and other regions [68]. Specifically, connections between the anterior cingulate cortex (ACC), an area involved in attention, mood, and reward processing are affected, leading to significant deficits in these aspects of cognitive control [69]. The DLPFC is therefore a plausible target for NIBS, as it can target several remote brain regions involved in the meso-cortico-limbic reward network. The connection between the ACC and DLPFC offers perhaps the most direct target for modulating prominent circuitry implicated in anxiety, because such individuals have reduced ability to assess positive reward and focus more on negative attention. PTSD in particular has key targets for NIBS which include the hippocampus, amygdala, and mPFC [70].

#### Transcranial magnetic stimulation

Within anxiety and trauma-related disorders, TMS has been most thoroughly applied to treat PTSD, with several meta analyses and systematic reviews supporting efficacy [71–73]. TMS has demonstrated robust overall effects for both PTSD and GAD, mostly through the application of high-frequency stimulation (10–20 Hz) over the right DLPFC [42, 70]. A few studies demonstrated that high-frequency rTMS yielded greater benefit for PTSD and GAD symptoms than lower frequencies [74–76]. This deviates from primary indications of rTMS involving low-frequency treatment for MDD and MDD with anxiety features. However, not all studies have found positive results from rTMS in PTSD. Particularly, a large RCT examining veterans with MDD found a higher rate of treatment-resistance to rTMS in those with comorbid PTSD [77]. This may be attributed to a limitation of rTMS in that it is only able to address dysfunction in one circuit (e.g., DLPFC to subgenual cingulate cortex (SGC)) at a time, and the pathology in PTSD involves multiple aberrant connectivity pathways. Notably, NIBS may be useful for modulating activity of the amygdala which is often hyperactive in anxiety disorders (particularly PTSD). While only a limited number of studies have tested NIBS in this context, they demonstrate success in regulating networks that include the amygdala and mPFC, which may transfer to pathologies of these regions [70, 78]. Similar samples demonstrate greater treatment benefit from ECT than rTMS; however, ECT is generally contraindicated in anxiety disorders without depression. Thus, replication of these findings is necessary. Research is still in developmental stages, but it is plausible that an increase in the number of stimulation sessions could lead to longer lasting and greater improvements as seen in other pathologies. Other anxiety disorders such as PD and social phobia remain largely understudied. While pilot studies do exist, conclusions cannot be drawn due to the limited literature and heterogeneity of trial parameters [79, 80].

#### Transcranial direct current stimulation

There are few published controlled studies evaluating tDCS in the context of trauma, anxiety, or stress-related disorders. A systematic review of 11 studies discussed mixed results in modulating anxiety-related symptoms through tDCS [65]. Selected studies primarily implemented left DLPFC anodal stimulation and right DLPFC cathodal stimulation, exploring theorized imbalances between left and right DLPFC activity across anxiety disorders. While most studies found significant effects, some did not [65, 81]. In females with SAD (*N* = 19*)*, a single session of anodal tDCS to the left DLPFC led to a reduction in attentional bias for threat compared to sham [82]. Although underpowered and not yet replicated, this proof-of-concept study holds important implications for both mechanistic research and the future of tDCS in SAD. In a randomized single-blind pharmacotherapy and sham-controlled study for patients with GAD, repeated cathodal tDCS over the rPFC decreased depressive and worry symptoms, but did not significantly alter anxiety symptoms [83]. Other studies in GAD have found no significant improvement in anxiety, stress, or depression after five sessions of 2 mA anodal tDCS applied to the left DLPFC vs sham [81]. Due to inconclusive results, limited samples, and a lack of published studies, it is premature to endorse tDCS as a treatment for anxiety disorders, particularly considering the range of experimental parameters, heterogeneous symptoms, and diverse behavioral outcomes. Future research exploring this modality should include larger, properly controlled RCTs. Investigation into activation of subcortical brain areas beyond previously described targets also deserve consideration [65].

#### Magnetic seizure therapy

As a novel intervention, the majority of MST research has focused on treatment-resistant depression (TRD), and no explicit study of MST for anxiety disorders has been published to date. Exploration of ECT has also been extremely limited in its application to anxiety disorders, particularly as the field shifts to less invasive stimulation modalities. Due to shared commonalities of ECT and MST, the literature reveals that ECT potentially improved symptoms in individuals with comorbid PTSD and MDD [84]. However, due to the adverse effects and patient burden associated with ECT, other interventions are recommended. MST may be useful for highly treatment-resistant cases of anxiety disorders due to its ability to produce focal seizures capable of inducing therapeutic change, as seen in depressive disorders [85]. More research is needed to understand whether MST is an effective treatment for anxiety disorders.

### Obsessive-compulsive disorder

OCD is characterized by the presence of unwanted intrusive thoughts (obsessions) and compulsive behaviors that interfere with social and occupational functioning [86]. Prevalence of OCD in the US is approximated at 1.2% of adults, with significantly higher diagnoses in females (1.8%) than males (0.5%) [87]. Typical treatment for OCD relies heavily upon a unique form of CBT known as exposure and response prevention (ERP). SSRIs and other forms of medications are also frequently utilized. Because of the severity and functional impairment often seen in OCD, as well as the propensity for treatment resistance, NIBS are of interest. The primary target for OCD involves the cortico-striato-thalamo-cortical circuitry, with specific targets depending on the depth of penetration of the stimulation technique [88]. Identified areas include the right and left DLPFC, pre-supplementary motor area (pre-SMA), orbitofrontal cortex (OFC), ACC, and mPFC. Subcortical targets typically require a deeper stimulatory technique than most noninvasive modalities can provide. Therefore, cortical regions closer to the surface that connect to the relevant regions of interest and are involved in disrupted circuitry are primary targets. Notably, rTMS is FDA-approved for OCD and is actively implemented in clinical practice [89]. Therefore, to align with the objectives of this review, our focus extends beyond the FDA-approved protocol of TMS delivered to the DMPFC. Instead, we delve into alternative stimulation interventions, including variations of TMS, such as TBS, and explore additional brain areas, like the DLPFC, SMA, and OFC. MST and tDCS are still emerging areas for OCD, and thus the current literature is reviewed, including rTMS, to discuss the scope and trajectory of NIBS for OCD.

#### Transcranial magnetic stimulation

There are two FDA-approved rTMS protocols for OCD, which include: 1. bilateral high-frequency (20 Hz) deep TMS (dTMS) to the DMPFC and ACC using an H-shaped coil approved in 2018 and 2. bilateral high-frequency (20 Hz) rTMS over the left and right DMPFC using a double-cone coil approved as an adjunctive therapy in 2020 [89–91]. Notably, rTMS for OCD can be used “online” with concurrent psychotherapy; online rTMS involves stimulation at discrete time points while patients engage with a cognitive task [20].

Exploration of rTMS for OCD continues to refine existing parameters to optimize patient outcomes. Low-frequency (1 Hz) rTMS administered to the right or left DLPFC appears superior to sham for the relief of OCD symptoms across several clinical trials [92, 93]. However, high-frequency rTMS to the DLPFC generally has not produced significant differences between active and sham groups in OCD [47]. With inconsistent results targeting the DLPFC, studies have explored other cortical regions, including the dorsomedial prefrontal cortex (DMPFC), OFC and SMA. Active high-frequency TMS targeting deeper structures, particularly the ACC, in patients with OCD has yielded significant reductions in symptom severity with effects persisting up to one-month [50]. A meta-analysis surveying 10 RCTs (*N* = 282) corroborated efficacy of active LF-rTMS over sham delivered to either the DLPFC or pre-SMA in treating OCD (35% vs 13% response rate respectively) [94]. Regions other than the DLPFC such as the OFC and SMA are other promising targets in ameliorating OCD [51]. Notable efficacy in non-DLPFC sites has been attributed to low-frequency inhibition of hyperactivity within these regions– an irregularity known to contribute to response control deficiencies and to suppress irrelevant information such as intrusive thoughts, impulses, or images. A meta-analysis analyzing the efficacy of various rTMS targets in treating OCD across 18 RCTs (*N* = 484) found low-frequency stimulation of the SMA yielded the greatest improvements in symptoms [52]. Interestingly, and in contrast to a previous meta-analysis, they found RCTs targeting the OFC to be non-significant in their efficacy [52]. Such inconsistent results necessitate further replication, but evidence suggests refinement of TMS may provide an effective supplemental treatment modality for OCD.

A newer form of rTMS, TBS, which utilizes bursts or high-frequency magnetic stimulation that can either be applied continuously (cTBS) or intermittently (iTBS), shows early promise for OCD intervention. Examination of cTBS as an adjunctive treatment for OCD has yielded encouraging outcomes, as evinced by Mukherjee et al., wherein neuronavigated accelerated cTBS led to a significant reduction in OCD symptoms [95]. Furthermore, two separate randomized trials employing cTBS bilaterally over the SMA resulted in significant alleviation of OCD symptoms [96, 97]. The evolving body of evidence suggests that the refined application of TMS, including innovative strategies such as TBS, and the strategic targeting of brain regions such as the SMA, may constitute an effective ancillary treatment approach for OCD.

#### Transcranial direct current stimulation

A limited number of open-label and controlled studies have explored tDCS for the treatment of OCD. A systematic review identifying 12 tDCS treatment studies for OCD (*N* = 77) found the following reductions in obsessive-compulsive symptoms: 26% (10 sessions left OFC cathode/right cerebello-occipital anode), 80% (20 sessions right OFC cathode/supplementary motor cortex anode), 30% (10 sessions right deltoid cathode/supplementary cortex anode), 64% (10 sessions left OFC cathode/right occipital anode), and 22% (20 sessions right DLPFC cathode/left DLPFC anode in conjunction with sertraline) [86]. These studies additionally reported decreases in related symptomology and comorbidity, such as depression and anxiety. However, findings were limited as several of the above reports were derived from individual case reports. Investigations of tDCS in OCD with and without pharmacotherapy have assessed a range of primary outcome measures, including decision-making [86]. Notable limitations to these reviewed studies include lack of sham-control comparison groups, small sample sizes, and wide ranges of parameters and regions of interest (ROIs). Although the few existing studies did find clinically meaningful results (at least 35% reduction in reported symptoms), the long-term effects, specific parameters, and ROIs must be carefully studied prior to endorsing tDCS as a treatment for OCD [88, 98].

#### Magnetic seizure therapy

Interest in administering MST for OCD largely stems from a collection of smaller studies suggesting ECT can be a viable option for highly treatment-resistant individuals [99]. Treatment-resistant OCD is often treated with deep brain stimulation (DBS) or in severe cases, ablative neurosurgeries. MST may offer a less invasive opportunity to resolve stubborn symptoms. Only one pilot study of MST delivered over the frontal cortex in treatment-resistant OCD has been published, reporting no benefit in a small patient sample [63]. Notably, depressive symptoms did not subside, possibly speaking to the difficulty in treating individuals with a greater number of comorbidities. Other studies of MST and OCD should ensue after new circuitry targets are identified.

### Borderline personality disorder

BPD is defined by a pervasive pattern of volatility in interpersonal relationships, identity, affects, suicidality and considerable impulsivity [100]. Impulsivity entails a complex collection of symptoms, such as self-injurious behaviors, substance abuse, and other high-risk behaviors. Prevalence of BPD in the general population is estimated at 1.6%, with a lifetime occurrence of 5.9% [101]. BPD is notoriously difficult to treat. Psychiatrists primarily incorporate psychotherapy and in some cases adjunctive psychotropic medications. BPD may benefit from NIBS to reach patients unresponsive to current treatment options and improve outcomes for those who do respond. Imaging studies show that BPD symptoms relate to disruptions in the frontolimbic network, specifically an hyperactive limbic system as well as reduced activity in the prefrontal regions [100, 102–104]. The frontolimbic network includes cortical regions implicated in regulatory control and limbic regions associated with emotion processing [100]. These relevant cortical regions include the ACC, OFC, and DLPFC.

#### Transcranial magnetic stimulation

There is growing evidence suggesting rTMS administered to the right or left DLPFC can reduce symptoms in BPD [55]. However, a lack of double-blind RCTs with sufficiently large sample sizes investigating therapeutic utility in this clinical population limits conclusions. Additionally, rTMS protocols for BPD are highly variable in terms of treatment parameters, including a wide range of stimulation frequencies (1–20 Hz or iTBS), a range of stimulation intensities (80–120%), and various targets (right or left DLPFC, bilateral DMPFC) [55]. Current studies of BPD primarily implement rTMS as a supplementary therapy for ongoing pharmacological and psychological treatments. A systematic review and meta-analysis by Yang et al. [105] found preliminary evidence for the ability of rTMS to modulate motor and temporal impulsivity in healthy adults, providing insights into possible mechanistic circuits underlying clinical populations with pathologized impulsivity, such as BPD [56]. Other reviews suggest rTMS could regulate and modulate various relevant characteristics associated with BPD, including emotion regulation, decision-making, and empathy [57, 58]. Preliminary results of double-blind RCTs involving right (1 Hz) or left (5 Hz) rTMS delivered to the DLPFC in BPD with comorbid mood disorders, show promise for improvements in depressive and BPD symptoms [106]. Findings were particularly significant in emotional instability and awareness, abandonment, impulsivity, paranoid ideation, and negative affect domains. Pilot studies also point to efficacy of rTMS targeting the DMPFC in treating MDD in BPD, with limited success in treating BPD symptoms themselves [107]. Overall, rTMS appears to be well-tolerated in individuals with BPD, but efficacy is largely unclear [108]. Longitudinal studies and additional RCTs will be valuable for determining acceptable ROIs and treatment outcomes.

#### Transcranial direct current stimulation

Only four RCTs investigating tDCS for BPD have been published to date [100]. In two of these studies electrodes were placed on the PFC with either bilateral anodal right or left DLPFC stimulation, and cathodal stimulation to the contralateral prefrontal area [109, 110]. The other two studies delivered unilateral stimulation with anodal right or left DLPFC and an extracephalic reference electrode over the mastoid [111, 112]. The variability in outcome measures included across these studies, such as emotion dysregulation, executive functioning, impulsivity, and stress-related dissociative states, make idirect comparisons of these studies difficult. Bilateral prefrontal stimulation appeared to improve impulsivity [109, 110], however other targeted clinical features revealed mixed results [100]. This is likely attributed to a wide range of employed parameters, particularly in the timing of sessions. For future exploration, it is recommended to expand upon existing findings and replicate anodal stimulation of the right DLPFC, as well as examine additional clinical features associated with BPD.

#### Magnetic seizure therapy

Currently, limited research has been conducted on the efficacy of MST for BPD. However, due to a strong prevalence of suicidality in individuals with BPD, there is a strong rationale for exploring novel treatments for BPD, and a clinical trial is in progress [113]. Encouragingly, a recent feasibility trial investigated the combination of MST and dialectical behavioral therapy (DBT) for patients with BPD and comorbid TRD found success with implementation [114]. While this study included only a limited sample (*N* = 19), findings suggested that conjoined DBT and MST were associated with significant reductions in depressive and BPD interpersonal symptom severity, as well as suicidal ideation. However, effects were not maintained at four-month follow-up. In studies of suicidality in MDD, MST has demonstrated significant benefit, with remission rates as high as 47% [115, 116]. The DLPFC has been suggested as a primary target due to frequent disruptions in related circuitry. ECT studies have revealed that individuals with MDD and comorbid BPD have greater risk of non-remission than those without BPD [117, 118]. However, the main targets were related to MDD and thus more precise targeting for BPD may yield different outcomes.

### Emerging modalities

Transcranial focused ultrasound (tFUS) and transcranial alternating current stimulation (tACS) are novel approaches for treatment of non-affective mood disorders. Decades of animal studies have suggested various areas of the central nervous system can be modulated by a tFUS with effects that persist for several hours post intervention [119]. Specific disorders have been modeled in animals, such as schizophrenia, indicating low-intensity pulsed ultrasound daily for five days induced significant changes in rodent models [120]. tFUS in human subjects is less explored, but preliminary evidence of somatosensory cortex, motor cortex, and thalamic stimulation show promise in modulating neural activity [121–124]. Notably, tFUS possesses higher spatial resolution capable of reaching deeper neural structures than magnetic and electric NIBS. This may allow for different structures to be more precisely targeted. Thus far, the few studies implementing tFUS have been safe and well-tolerated, reporting no serious adverse effects [119].

Applications of tFUS for psychiatric purposes have demonstrated early success in depression, OCD, and chronic pain [125–129]. In humans, tFUS has also been found to significantly improve depression syndromes and normalize functional connectivity measures, particularly between the salience and ventromedial networks in patients with Alzheimer’s disease [125]. Low-intensity tFUS trials for depression and anxiety are currently open and recruiting [130]. Rigorous research is necessary to clarify the dosing, efficacy, and potential utility of tFUS for the treatment of psychiatric disorders.

There is growing support for the feasibility of tACS as a clinical tool capable of altering behavioral outcomes. Initial results investigating tACS in psychiatric populations support its ability to reset disturbed brain oscillations [131]. However, much of the existing literature is dependent upon case reports with few published RCTs. Among these, seven publications have examined tACS in patients with schizophrenia [131–135]. The majority of these demonstrated positive effects in outcomes ranging from working memory to auditory hallucinations. Yet, there were no significant clinical outcomes for change in negative and positive symptoms despite certain modifications in neural oscillatory patterns. More studies will enlighten relevant mechanisms of action. A handful of studies in depression and OCD have also shown improvement in symptoms in small sample sizes. While case reports can be useful for individual patient outcomes, feasibility, and highlighting areas of promise, larger studies and refined protocols will be paramount to clarifying the future of tACS for psychiatric conditions.

## Discussion

Noninvasive brain stimulation techniques offer promising results for treating nonaffective psychiatric disorders, but more research is required to better understand optimal parameters and localize target brain regions. Across stimulation modalities, mechanisms are largely unknown, but links to neuroplastic changes are compelling. Supplementary Table 1 summarizes the current status of these modalities. These neuroplastic changes vary in excitation or inhibition depending on treatment parameters and targeted regions. Connectivity alterations from neuromodulatory currents are thought to enact either LTP or LTD [16]. Brain regions and neural circuits associated with disruptions in certain disorders have been found to be valuable treatment targets, such as hyper-connectivity between DLPFC and the TPJ in psychosis, disruptions between ACC and DLPFC in anxiety disorders, cortico-striato-thalamo-cortical circuitry abnormalities in OCD, and aberrant connectivity in the frontolimbic network in BPD. Exploring stimulation of known neurobiological abnormalities in other disorders may be beneficial for treating disorders beyond those discussed here. Novel forms of stimulation are gaining traction, such as the use of transcranial ultrasound for the treatment of TRD. Preliminary evidence are promising, but investigations are still in early stages.

Of the reviewed literature, all included disorders present as potential candidates for various noninvasive brain stimulation treatments. Evidence suggests that positive symptoms in psychotic disorders, schizophrenia in particular, prove challenging to treat with NIBS. While some evidence exists for reducing positive symptoms with rTMS, collectively NIBS do not appear to effectively and consistently reduce positive symptom severity [42]. Both tDCS and MST have received less exploration. tDCS for psychosis has yielded mixed results, likely attributed to a wide range of parameters employed. Much of the literature investigating MST for psychosis is limited to pilot and feasibility studies, which have found significant reductions in symptoms and improvements in quality of life. However, treatment adherence was a notable problem interfering with study completion and diminishing sample size. Alternatively, negative symptoms in psychosis appear to respond effectively to both TMS and tDCS protocols, with medium effect sizes. Existing studies also support rTMS for the treatment of GAD. Findings for studies administering rTMS for PTSD are mixed, and inferior to ECT in terms of symptom reduction. The majority of tDCS studies for anxiety or stress-related disorders (including GAD, PTSD, and SAD) found significant improvements in symptoms. With the exception of OCD, MST has not been studied for anxiety disorders to date, but may present a viable therapeutic tool based on success with mood disorders. As an FDA-approved treatment for OCD, rTMS is already implemented into clinical practice as an adjunctive therapy. tDCS studies for OCD were extremely limited, but did highlight promising results and the need for future research. For BPD, rTMS appears to alleviate impulsivity and emotional dysregulation. tDCS and MST cannot yet be recommended due to a dearth of studies and mixed evidence. Overall, rTMS demonstrated significant effects and utility across all included disorders. tDCS presented mixed results with areas of promise, and MST literature is currently limited.

The existing NIBS literature provides substantial evidence for brain stimulation in the reduction of depressive symptoms and treatment of mood disorders, particularly MDD. Expanding application of stimulation modalities to different disorders by targeting underlying circuitry disruptions may be critical for future interventions. Further, optimizing frequency, duration, number of sessions, and ROI localization, can provide greater benefits and patient outcomes. The development of individualized protocols has additionally gained attention; incorporating complementary technologies such as fMRI and EEG allows for individually curated treatment regimens. Although rTMS is an approved treatment for depression, other forms of stimulation such as tDCS show promise in symptom reductions and require further investigation. MST is the newest and least studied NIBS technique, yet early stages support its clinical utility. However, significant limitations are important to address, such as limited RCTs across modalities, small sample sizes, lack of sham-controlled comparison groups, and few published studies in certain conditions and disorders. Further, ethical concerns are important to consider, such as potential restriction of access to effective treatments in sham comparison trials.

Opportunities for increasing efficacy for NIBS should be rooted in individualizing treatment parameters, clarifying discrepancies between delivered dose and received dose, and refining targeting specificity. Several methods for individualized treatment have been proposed and are in the process of being examined. These include utilizing structural imaging such as MRI to target the individual’s identified brain structures of interest more precisely. Further, electrical-field modeling illustrates the anticipated depth and intensity of current stimulation within the individual’s brain, allowing for optimization of targeted coordinates and angles. Ensuring the appropriate regions of interests are in fact receiving the induced current is vital for ensuring optimal treatment outcomes and informing greater knowledge of underlying neurocircuitry. Refining treatment parameters will potentially lead to increases in efficacy across disorders and domains. These disorders persist because they are difficult to treat. However, such interventions as described can regulate disrupted activity and cortical connections. Before this can be achieved, it is necessary to refine treatment parameters and conduct further research.

## Conclusion

The field of NIBS presents an array of promising therapeutic approaches for nonaffective psychiatric disorders. The proven efficacy of TMS, tDCS, and MST for mood disorders underscores the vast potential of NIBS. Meanwhile, emergent techniques such as tFUS and tACS extend the frontier of neuromodulation possibilities. However, significant scientific and clinical challenges persist. Standardized treatment parameters, optimal target brain regions, and the underlying mechanisms of NIBS modalities require further elucidation. Additionally, methodological inconsistencies, insufficient RCTs, small sample sizes, and the paucity of studies for specific disorders constitute substantial barriers to progress. Pairing NIBS with advanced neuroimaging technologies like fMRI and EEG represents a promising strategy to optimize treatment parameters and develop individualized protocols based on patient-specific neuroanatomy and brain function. This integration could significantly enhance treatment outcomes. Furthermore, the pressing need to generate rigorous, high-quality scientific evidence to inform standardized treatment guidelines cannot be overstated. Such evidence is pivotal for broadening the regulatory approval of NIBS for various psychiatric disorders. In conclusion, although the prospect of NIBS in psychiatry is promising, the imperative to expedite scientific investigation remains. Doing so will not only deepen our understanding of these novel therapies but also streamline the development of standardized treatment protocols, catalyzing the clinical translation of these groundbreaking neuromodulatory approaches.

### Supplementary information

Supplementary Table 1
