# Supplementary material for: Pre-clinical indications of brain stimulation treatments for non-affective psychiatric disorders, a status update
Source: Transl Psychiatry. 2023 Dec 14;13:390. doi: 10.1038/s41398-023-02673-2 (PMC10721798; doi:10.1038/s41398-023-02673-2)
Supplement: Supplementary file 1 — Supplementary Table 1 [file 41398_2023_2673_MOESM1_ESM.docx]

**Supplementary Table 1: Emerging NIBS Status and Targets**

|  | **Mechanism of action** | **Primary Target for Psychosis** | **Target for Anxiety disorders** | **Target for OCD** | **Approval stage** | **Advantages** | **Disadvantages** |
| --- | --- | --- | --- | --- | --- | --- | --- |
| **tDCS** | Electric current | DLPFC, TPC | DLPFC, DMPFC, VLPFC | OFC, pre-SMA, DLPFC | Undergoing approval process | Portability, No serious adverse effects, high tolerability | Mixed results |
| **rTMS** | Electric current induced by electromagnetic field | DLPFC, TPJ, TPC | DLPFC, motor cortex | OFC, SMA, DLPFC | FDA-approved for clinical use | Effective in individuals with treatment-resistance, no cognitive impairment | Expensive, risk of seizure (very low), substantial time course |
| **MST** | Delivered electrical current induces therapeutic seizure | Frontal Cortex | Frontal Cortex | Frontal Cortex | Investigational | Similar success as ECT with less side effects | Requires sedation, currently does not have standardized procedures |
| **tFUS** | Non-destructive mechanism pressure on cellular membranes and ion channels | Mediodorsal Thalamus | Prefrontal Cortex, subgenual cingulate (Brodman’s area 25) | Anterior limb of the internal capsule and anterior cingulate gyrus | Investigational | Higher spatial resolution that can target deeper structures | Limited evidence, early stages |
| **tACS** | Electric current | Medial prefrontal region | Occipitoparietal sites | OFC | Investigational | Portability, No serious adverse effects, high tolerability | Limited evidence for utility in psychiatric disorders |
